# A Solitary Sigmoid Perineurioma in an Otherwise Healthy 30-Year-Old Male

**DOI:** 10.7759/cureus.15104

**Published:** 2021-05-18

**Authors:** Sara Kamionkowski, Abdulfatah Issak, Claire Zhang, Yan Wang, Annette Kyprianou

**Affiliations:** 1 Internal Medicine, MetroHealth Medical Center, Cleveland, USA; 2 Gastroenterology and Hepatology, MetroHealth Medical Center, Cleveland, USA; 3 Pathology, MetroHealth Medical Center, Cleveland, USA

**Keywords:** perineurioma, polyp, colonoscopy, colon cancer, peripheral nerve sheath tumor

## Abstract

Colorectal perineuriomas are rare benign fibroblastic polyps of the colon found on colonoscopy and usually present as a sessile polyp distal to the splenic flexure. We report a case of sessile sigmoid perineurioma in a young healthy male. He presented with chronic constipation and underwent colonoscopy, which showed a 3-4 mm sessile polyp in the sigmoid colon. Biopsy results were significant for a perineurioma. These polyps are peripheral nerve sheath tumors composed of bland spindle cells with ovoid nuclei in a whorling appearance. The differential diagnosis of these nerve sheath tumors includes ganglioneuromas, schwannomas, neuromas, neurofibroma, or Schwann cell hamartomas, and gastrointestinal stromal tumor (GIST). While these polyps are regarded as benign, it is prudent to rule out other tumors that have malignant potential.

## Introduction

Colorectal perineuriomas are uncommon benign fibroblastic polyps of the colon that have been better described histologically in the last 20 years. They comprise only about 0.1% to 0.46% of colonic polyps found on routine colonoscopy and generally present as a sessile polyp distal to the splenic flexure. We report a case of sessile sigmoid perineurioma in a young healthy male. This case report was previously presented at the American College of Gastroenterology (ACG) ePoster Hall (Poster: Kamionkowski S, Issak A, Zhang C, Kyprianou A. A Solitary Sigmoid Perineurioma in an Otherwise Healthy 30-Year-Old Male. ACG 2020 Virtual; October 23-28, 2020).

## Case presentation

**Figure 1:**
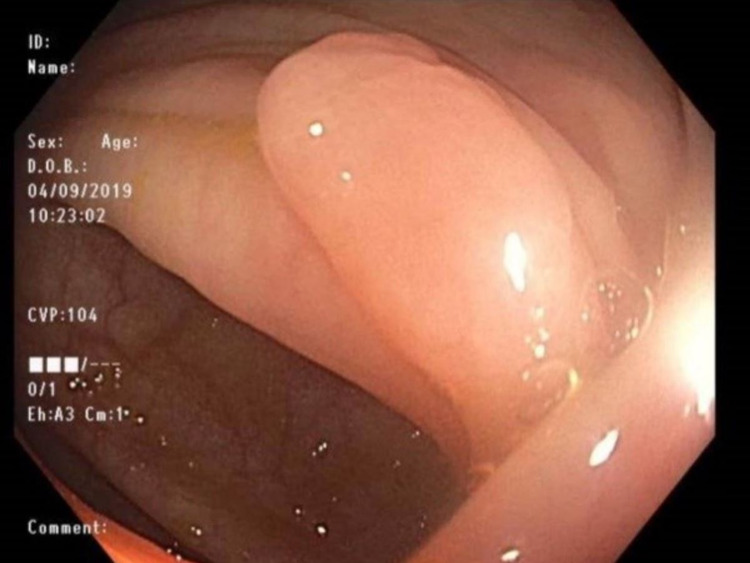
4-mm sessile polyp in sigmoid colon

**Figure 2:**
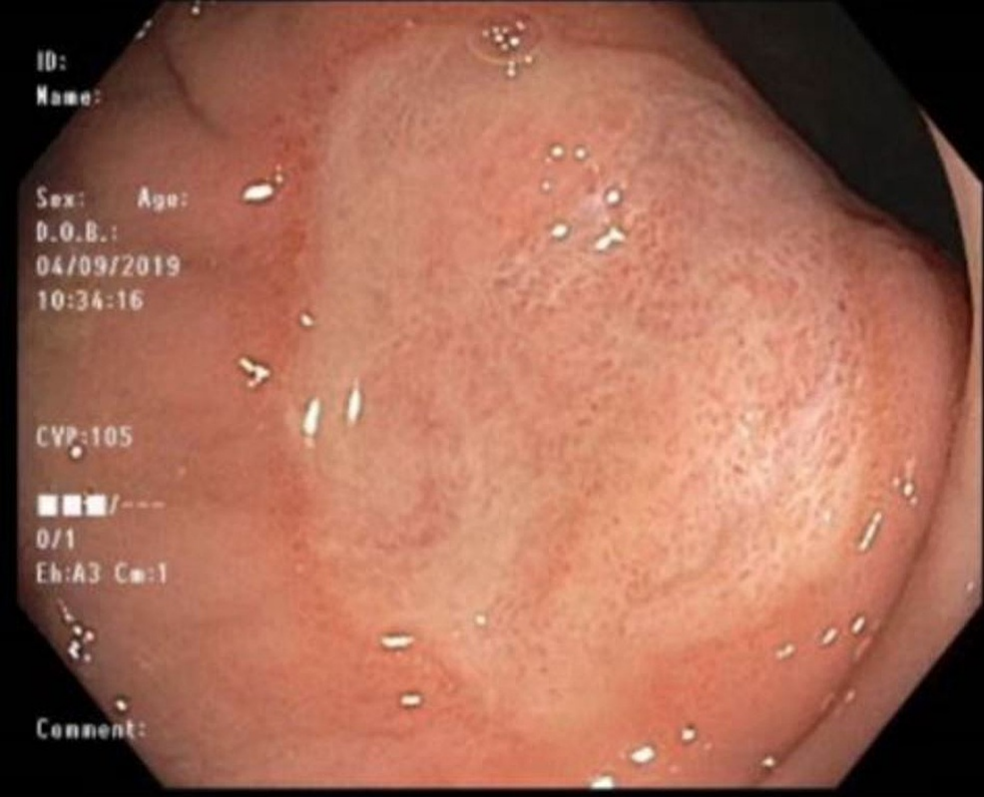
3 x 3 cm stercoral rectal ulcer

**Figure 3:**
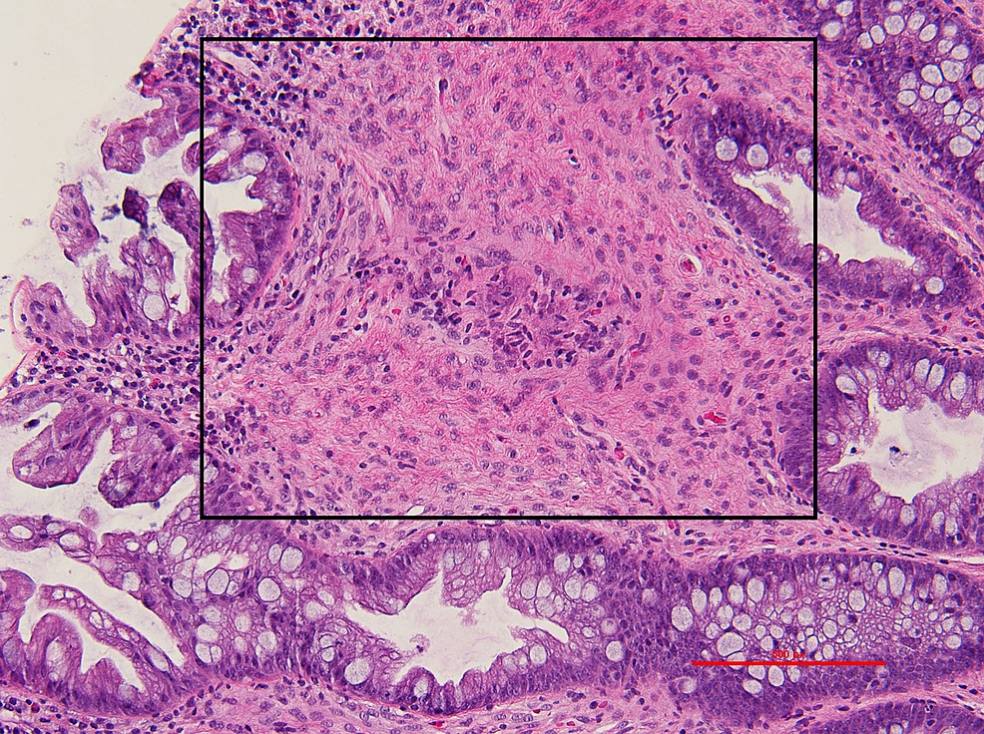
Pathology of sessile sigmoid polyp biopsy. A poorly circumscribed spindle cell lesion confined to the mucosa is highlighted by the black rectangle; hematoxylin-eosin (H&E), photographs using a 20x objective (x200 total magnification)

**Figure 4:**
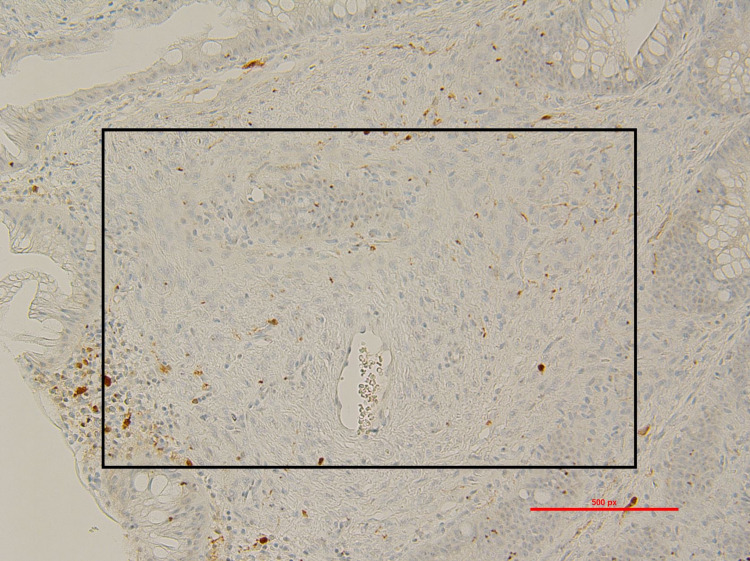
S100 immunohistochemistry of sessile sigmoid polyp. Neoplastic cells in this spindle cell lesion are negative for S100 stain which are highlighted by the black rectangle; photographs using a 20x objective (x200 total magnification)

A 30-year-old male with a history significant for chronic constipation since childhood presented for his third diagnostic colonoscopy. He has a family history significant for a paternal uncle who was diagnosed with colon cancer at age 40. The patient suffered severe constipation since childhood despite the use of multiple over-the-counter medications including polyethylene glycol, senna, and docusate sodium. This history prompted his initial colonoscopic examination at age 18. His initial colonoscopy had shown three polyps. Unfortunately, histology of these polyps was unknown as the procedure was performed at an outside hospital. At presentation to our gastroenterology clinic, he still complained of mild constipation with occasional scant blood noted on stools. He denied weight loss, abdominal pain, nausea, and vomiting. His physical exam was unrevealing without any abdominal or distention, rashes, or skin lesions. He underwent colonoscopy; however, it was deemed incomplete due to poor prep in the right colon. His colonoscopy showed a 3-4 mm sessile polyp in the sigmoid colon that was removed with cold snare (Figure 1). The rectum showed a 3 x 3 cm patch with biopsy results significant for fragments of an ulcer, believed to be a solitary rectal ulcer in the setting of his chronic constipation (Figure 2). The pathology report of the sigmoid sessile polyp was significant for a mucosal perineurioma. Histological examination showed a poorly circumscribed proliferation of uniform, plump spindled cells in the lamina, spindled cells with eosinophilic cytoplasm, indistinct cell borders and ovoid to spindled nuclei with inconspicuous nucleoli, absence of ganglion cells, pleomorphism, mitotic figures and necrosis (Figure 3). Immunohistochemical staining showed the spindle cell population to be negative for S100, which ruled out Schwann cell hamartoma and ganglioneuroma, hence, confirming the diagnosis of perineurioma (Figure 4). The patient was counseled to follow up in one year for a repeat colonoscopy due to poor prep.

## Discussion

Perineuriomas (also called benign fibroblastic polyps) are peripheral nerve sheath tumors, similar to neurofibromas, schwannomas, and ganglioneuromas. They are most commonly seen on the skin as flesh-colored papules [1]. They are not typically known to be associated with gastrointestinal symptoms and are typically found incidentally on colonoscopy with a benign course [2] Histologically they are composed of bland spindle cells with ovoid nuclei in a whorling appearance. Nuclear pleomorphism, mitotic activity, and necrosis are often absent [3]. Immunohistochemistry is often strongly positive for glucose transporter-1 (GLUT-1), claudin-1, and less specifically for epithelial membrane antigen (EMA) [4]. BRAF V600E mutations are found in about 63% of these polyps, but notably, only ones that presented with serrated epithelium [5].

Perineuriomas comprise only about 0.1% to 0.46% of colonic polyps found on colonoscopy and are often found incidentally in asymptomatic patients. Around 150 case reports of colonic perineuriomas have been published and most had presented as small sessile polyps [6]. They can present as pedunculated or serrated morphology. The two major types include mucosal or submucosal (intramural), and the differential diagnosis depends on where the perineurioma is located. If it is mucosal, the differential may include ganglioneuromas, schwannomas, neuromas, neurofibroma, or Schwann cell hamartomas; if submucosal, it may be schwannoma versus a gastrointestinal stromal tumor (GIST). GIST is one of the most important tumors that need to be ruled out as it has malignant potential. GIST, however, tends to be significantly larger than perineuriomas with an average size of 20 mm whereas perineuriomas are much smaller, around 4 mm [2,7]. Histologically, GIST may show hyperchromasia and mitotic activity [2,7]. To date, there have not been any formal case control or cohort studies to assess the risk of malignant potential or recurrence especially in those polyps that are positive for BRAF V600E mutations. Similarly, ideal interval time for follow-up surveillance colonoscopy in patients with colonic perineuriomas has not been established.

## Conclusions

While these polyps are currently regarded as benign, it is prudent to rule out other tumors that have malignant potential such as GIST. This case report adds to growing literature highlighting incidences of colonic perineuriomas in the last 20 years.
